# Supplementary material for: Increasing Care Partners’ Capacity for Supporting Individuals Living with Dementia Through Bravo Zulu: Achieving Excellence in Relationship-Centered Dementia Care
Source: Int J Environ Res Public Health. 2025 Jun 20;22(7):970. doi: 10.3390/ijerph22070970 (PMC12294183; doi:10.3390/ijerph22070970)
Supplement: Supplementary file 1 [file ijerph-22-00970-s001.zip › ijerph-3672484-Supplementary Document-rev 06 19 2025.pdf]

Supplementary Document: Increasing Care Partners' Capacity for Supporting Individuals Living with Dementia Through Bravo Zulu: Achieving Excellence in Relationship-Centered Dementia Care

Table S1. Overview of Bravo Zulu Modules, Learning Objectives, Content, and Associated Learning Tasks

Module*	Learning Objective	Content Outline	Learning Tasks
Module V<br><br>Valuing Personhood, Relationships, and Culture	Participants will explore how valuing personhood, relationships, and culture enhances dementia care, and will learn to apply cultural humility by honoring each individual's life story, values, traditions, and preferences.	Valuing Personhood<br>- Recognizing the need for person-centeredness<br>- Appreciating core values<br>- Honoring life history, rituals, traditions, and preferences<br>Valuing Relationships<br>- Valuing and supporting important relationships<br>- Exploring the six senses of relationship-centered care<br>- Working in partnership with elders and other care partners<br>Valuing Culture<br>- Appreciating the importance of cultural competence and cultural humility<br>- Understanding military culture	- Reflective journaling<br>- Peer sharing<br>- Group discussion<br>- Values clarification<br>- Scenario-based problem solving<br>- Perspective taking<br>- Relationship mapping<br>- Relationship-centered planning<br>- Storytelling analysis<br>- Self/environmental assessment
Module I<br><br>Treating People as Unique Individuals	Participants will learn to develop individualized approaches to supporting well-being that reflect each person's unique identity, life experiences, preferences, and strengths, while systematically exploring multiple, interconnected dimensions of well-being to promote autonomy, meaning, and connection.	Supporting each person's well-being<br>- Exploring Kitwood's Enriched Model of Dementia<br>- Applying Eden Alternative's Seven Domains of Well-Being<br>Nurturing identity and connectedness<br>- Understanding each person's life story and significant events<br>- Reflecting on *15 Things Veterans Want You to Know*<br>Fostering security and autonomy<br>- Fostering a sense of home through personal possessions<br>- Supporting individual preferences<br>Promoting meaning, growth, and joy<br>- Engaging in meaningful and purposeful activities<br>- Living and celebrating life through leisure	- Group discussion<br>- Guided lived experience interview<br>- Scenario-based problem solving<br>- Perspective taking<br>- Storytelling analysis<br>- Values clarification<br>- Self/environmental assessment<br>- Life story exploration<br>- Narrative-based group reflection<br>- Leisure mapping<br>- Personal meaning inventory

Module*	Learning Objective	Content Outline	Learning Tasks
Module P<br><br>Looking at the World from the Perspective of the Person	Participants will explore proactive approaches to supporting well-being and reframe so-called "dementia-related behaviors" as communication, learning to decode and respond to expressions of distress or unmet needs by seeking to understand the perspective of the person living with dementia.	Critiquing the biomedical view of dementia<br>- Rethinking so-called "dementia-related behaviors"<br>- Shifting from pathologizing behaviors to understanding and validating personal expressions, actions, and reactions<br>Taking a proactive approach to supporting well-being<br>- Assessing unmet needs and positive support strategies<br>- Identifying common beliefs and practices that threaten well-being<br>Perceiving different realities<br>- Understanding other factors that may shift the way a person perceives and experiences the world, including prior military stressors, trauma, sensory changes, and mental illness<br>- Asking about and supporting past experiences, while recognizing when to refer to a mental health professional	- Storytelling analysis<br>- Group discussion<br>- Peer sharing<br>- Critical comparison and reflective dialogue<br>- Perspective taking<br>- Case study reflection<br>- Values clarification<br>- Strengths-based assessment<br>- Critical reflection<br>- Relationship-centered planning<br>- Guided self-reflection on labels and bias<br>- Well-being assessment
Module S<br><br>Providing a Positive and Supportive Social Environment	Participants will practice skills for interacting with people living with dementia and learn to foster a supportive social environment that promotes meaningful inclusion and sustained community engagement.	Understanding how dementia affects communication<br>- Communicating effectively with people experiencing expressive or receptive aphasia<br>Improving communication for connection and understanding<br>- Identifying negative social interactions<br>- Practicing positive social interactions<br>Supporting opportunities for continued engagement in life<br>- Identifying meaningful leisure experiences<br>- Exploring engagement possibilities, including veteran-centric activities<br>- Building bridges for continued community engagement and citizenship	- Group discussion<br>- Interpreting emotional communication exercise<br>- Non-verbal communication role play<br>- Observation and reflection on care interaction videos<br>- Photo-based group dialogue<br>- Applied brainstorming<br>- Perspective taking<br>- Storytelling analysis<br>- Engagement planning

*VIPS framework adapted from Brooker, 2007. [1]

Further Information

The training curriculum featured in this study was independently developed by Jennifer Carson and was not influenced or co-developed by the other co-authors. Additional information about the program is available at https://deerprogram.org/bravo-zulu/. For direct inquiries, please contact Dr. Jennifer Carson at the Dementia Engagement, Education, and Research (DEER) Program, School of Public Health, University of Nevada, Reno, NV 89557, USA. Email: jennifercarson@unr.edu.

1. Brooker, D. *Person-Centred Dementia Care: Making Services Better*. Jessica Kingsley Publishers: London, UK, 2007.
